# Targeting frizzleds with small-molecule compounds—back to square one or light at the end of the tunnel?

**DOI:** 10.1016/j.jbc.2026.111431

**Published:** 2026-04-08

**Authors:** Gunnar Schulte

**Affiliations:** Section of Receptor Biology & Signaling, Department Physiology & Pharmacology, Karolinska Institutet, Stockholm, Sweden

**Keywords:** class F GPCRs, drug discovery, Frizzleds, FZD, G protein-coupled receptor (GPCR), medicinal chemistry, negative allosteric modulators, WNT signaling

## Abstract

The Frizzled (FZD) family of G protein-coupled receptors (GPCRs) are central mediators of Wingless/Int-1 (WNT) signaling that regulates embryonic development, stem cell function, and tissue homeostasis. This makes FZDs attractive therapeutic targets, for example, in cancer and fibrotic diseases. However, developing small-molecule drugs targeting these class F GPCRs has been extraordinarily difficult. Recent breakthrough achievements demonstrate that small-molecule allosteric modulators can successfully target the FZD core. Despite low potency, recent data show that the central cavity of FZDs is indeed druggable and that negative allosteric modulators (NAMs) can block agonist-induced oncogenic β-catenin signaling. Importantly, intrinsic FZD conformational rearrangements are required for FZD activation and signal initiation, supporting an integrated dynamic GPCR activation model for FZDs, which builds the functional and molecular basis for NAM action. Future optimization efforts will improve compound affinity, potency, and achieve FZD or cluster selectivity to minimize unwanted effects on active stem cell niches, for example in epithelia and bone while advancing toward clinical applications.

Cell-to-cell communication is predominantly controlled by activation of specific cell surface receptors localized within the plasma membrane. Receptor-activating ligands are either secreted from (paracrine) or presented on the surface (cell contact) of neighbouring cells or presented as a humoral ligand with systemic effect (endocrine). Ligand-receptor interaction initiates conformational changes in the transmembrane receptor protein to activate diverse intracellular signal transduction events that alter cellular behaviour in a context-specific manner.

The Wingless/Int-1 (WNT) signaling system is an evolutionarily conserved, tightly regulated signaling network comprising multiple transmembrane receptors and secreted ligands that mediate central processes during embryonic development, stem cell regulation and tissue maintenance and regeneration (1). In mammals, the complex WNT signaling system is composed of nineteen different WNTs, secreted lipoglycoproteins that serve as primary ligands for the 10 Frizzled family receptors (FZD_1-10_).

FZDs, together with Smoothened (SMO) which mediates Hedgehog signaling, belong to the class F of G protein-coupled receptors (GPCRs) (2, 3, 4). Based on sequence homology, the 10 FZDs can be grouped into homology clusters: FZD_1, 2, 7_; FZD_3, 6_; FZD_4, 9, 10_; and FZD_5, 8_. Generally, the different FZDs behave more similar within a cluster than across. In addition to the main WNT receptors of the FZD family, WNTs interact also with single transmembrane domain spanning receptors, which act as co-receptors and which are important to specify the downstream signaling output. While LDL receptor-related protein five-sixths (LRP5/6) determine signaling towards the transcriptional regulator β-catenin, ROR1/2, RYK and PTK7 mediate signaling in a β-catenin-independent manner (5). Despite the diversity in the role of the WNT co-receptors, the central, FZD-interacting transducer protein Dishevelled (DVL) existing in three paralogs in mammals (DVL1, 2, 3) is positioned at the crossroads of all these avenues. Furthermore, FZDs also couple to heterotrimeric G proteins, thereby presenting a second line of signal transduction mutually exclusive of DVL coupling (6, 7, 8, 9, 10, 11, 12, 13, 14, 15, 16, 17, 18, 19, 20).

## FZD architecture in brief

**Figure 1:**
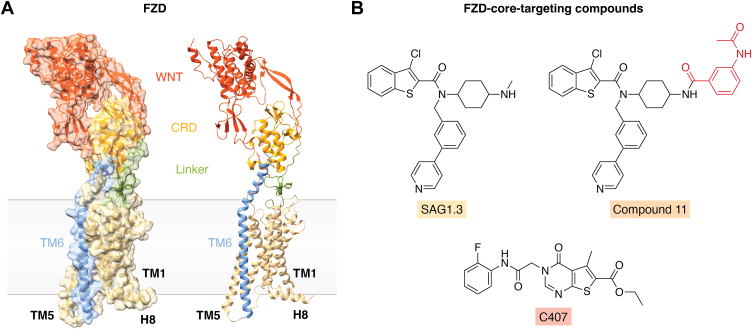
**FZDs and FZD-core-targeting compounds.***A*, depiction of the overall architecture of FZD family receptors. WNTs (*red*) interact with the cysteine-rich domain (CRD; *yellow*) of FZDs. The CRD is connected to the core through a linker domain (*green*), which contains disordered and ordered regions. The long TM6 (*blue*) of all FZDs except for FZD_4_ could exert a lever function contributing to an allosteric connection between the CRD and the transducer interface on the intracellular side (the model is a merge of PDB ID 6O3C and 4F0A; created with ChimeraX). *B*, structural depiction of SAG1.3, compound 11 and C407. The red part of compound 11 emphasizes the structural difference compared to SAG1.3. Structures were created in MolView (26, 70, 78).

Class F GPCRs have a peculiar architecture with a large, extracellular, globular cysteine-rich domain (CRD) at the N terminus (Fig. 1*A*). This domain harbors the primary, orthosteric binding site(s) for WNTs, which interact with the CRD through a protein-protein interface and through a combined protein-protein/protein-lipid interface, given the conserved lipidation of WNT proteins (21, 22). The CRD is linked to the receptor core by a linker region, which combines structured and unstructured, flexible elements. The seven transmembrane spanning core and the membrane-associated helix 8 (H8) are more reminiscent of a classical GPCR architecture and include family-wide conserved molecular switches important for receptor activation and transducer coupling (17, 19, 23, 24, 25). A distinct feature of class F GPCRs—with the exception of FZD_4_—is the long transmembrane helix 6 (TM6), which protrudes above the cell membrane for several helical turns, and which is of central importance to determine the architecture of the extracellular domains of the receptors including the extracellular loop 3 (ECL3), the linker domain and the CRD (3). Unfortunately, the mechanistic contribution of TM6 dynamics contributing to linking the CRD with the FZD-transducer interface allosterically is poorly resolved so far. Importantly, TM6 is in physical contact with the linker region and possibly the CRD (Fig. 1) and contains a conserved proline at position 6.43, which allows the helix to kink and to swing out thereby accommodating transducer coupling (18, 24, 26). Furthermore, it is well established that WNT binding to the CRD elicits downstream signaling, where a chain of events elicits dynamic alterations from WNT binding, CRD dynamics relative to the receptor core, core conformational rearrangements and receptor–transducer interface dynamics. Despite this overall concept of intramolecular signal transduction, it is less well understood how WNT binding to the CRD physically elicits information flow throughout the receptor molecule to communicate with the intracellular transducers and to initiate and specify an intracellular signal.

The analysis of the mechanistic chain of events from ligand binding to transducer coupling has been catalyzed by the development of a series of biophysical biosensors and live cell assessment of WNT-induced dynamic changes (3, 27). Bioluminescence resonance energy transfer (BRET)- and fluorescence-based, genetically encoded biosensors were used to dissect WNT-FZD binding (28, 29, 30), WNT-induced CRD dynamics (31), FZD core dynamics (32), FZD molecular switch mechanisms, and FZD-transducer interface dynamics (17, 19, 24, 25, 33), overall emphasizing receptor activation dynamic of FZDs. Despite the progress in this direction, it remains still poorly defined, what agonist-induced FZD activation indeed refers to, a concept that is obviously of intrinsic importance when discussing ways to target FZDs pharmacologically (3). Structural, pharmacological and biochemical evidence clearly underline that FZDs interact with transducers in constitutively active manner. Constitutive Gs coupling of FZD_7_ for example results in elevated cAMP levels (18, 34). Furthermore, DVL (or the minimal FZD-binding DEP domain of DVL) bind FZDs in a constitutive manner and a FZD_4_-DEP cryogenic electron microscopy (cryo-EM) structure revealed insights into structural details (26). However, biophysical, genetically encoded biosensors, revealed a WNT-induced structural rearrangement at the FZD-DVL (DEP) interface in comparison to constitutive FZD-DVL (DEP) recruitment, suggesting that the agonist-induced active state towards FZD-DVL (DEP) coupling remains to be resolved (25).

The overall established model for how the WNT signal is translated into the action of β-catenin as a transcriptional regulator is summarized in a signalosome model, where WNTs simultaneously bind to FZDs and LRP5/6. Forming a receptor complex and also higher order agglomerates of these complexes feeds into the inhibition of the β-catenin destruction complex, stabilizing cytosolic β-catenin and enabling its translocation to the nucleus, where it regulates T-cell factor/lymphoid enhancer-binding factor (TCF/LEF) transcription factors (35, 36, 37). Importantly, there is a widely accepted belief that the physical assembly of the signalosome, including FZD, LRP5/6 and intracellular adaptor proteins, rather than FZD protein dynamics, is driving signal initiation of the β-catenin pathway. This model, known as the signalosome model, neglects important mechanistic aspects of the process, including transmembrane allostery (long-range cooperativity between the extracellular ligand binding site and intracellular transducer interface), protein dynamics, and class A GPCR-like conformational changes in FZDs (38, 39). The importance of these processes involving “outside-in” information flow and pathway-selective allostery in—as encompassed in the dynamic FZD activation model—is supported, for example, by the argument that the CRD is required for constitutive FZD_7_-G protein coupling, whereas the CRD is not required to recruit DVL or interact with the isolated DVL DEP domain (18, 40).

While a certain controversy between the so-called signalosome model and the dynamic model exists in the field, the focus of this article on small-molecule compounds targeting FZDs inevitably argues that we have to redefine the depiction of WNT/FZD activation by integrating the signalosome model with the concepts of a GPCR-like allosteric transmembrane coupling between the orthosteric WNT binding site and the FZD-transducer interface as previously argued, suggested and shown (3, 16, 19, 25, 32, 39).

Why target FZDs pharmacologically with small molecules?

About 36% of all FDA approved drugs target GPCRs and 121 of the 362 non-sensory members (33%; often called conventional or "drug-targetable" GPCRs in contrast to the sensory GPCRs) of the GPCR superfamily are targeted (41). Since the discovery of FZDs as WNT receptors and their classification as members of the GPCR superfamily, the hypothesis emerged that FZDs are druggable (42, 43, 44). FZDs are intrinsically involved in embryonic development, tissue homeostasis, and regeneration (45). In addition, exaggerated WNT/FZD signaling is, for example, implicated in diverse forms of tumors or in fibrosis, where the pharmacological concept of antagonists, inverse agonists or negative allosteric modulators could potentially serve as treatments (46, 47). Less common are mutations compromising FZD function, such as in the hereditary eye disease familial exudative vitreoretinopathy, where impaired FZD_4_ signaling is linked to vascular defects in the retina, leading to impairment or loss of vision (48, 49). Here, loss of function of FZD_4_ demands positive compensation using agonists or positive allosteric modulators (PAMs), such as the recently developed FZD_4_-LRP5 bridging surrogates (50, 51, 52).

The development of FZD-targeting approaches during the last 10 years or so went mostly into the exploration of CRD-targeting antibodies (as antagonists) or so-called WNT surrogates artificially bringing FZDs and LRP5/6 into proximity to promote signaling for example in regenerative approaches (47, 52, 53, 54, 55, 56). Despite conceptual progress and therapy-relevant breakthroughs (50, 57), biologics come obviously with a series of caveats, such as poor cell and tissue permeability, high manufacturing costs, challenges in complex manufacture, limited stability connected to challenging storage logistics, the risk of immunogenicity, and administration difficulties with the requirement of intravenous or subcutaneous administration. While all these shortcomings can be managed if the therapeutic advantage is sufficiently large, small molecules have proven superior in many of the above-mentioned aspects if selectivity is sufficient and off-target effects are manageable or limited. In addition, the timing to search for FZD-targeting small molecules is now better than ever, with growing virtually screenable and synthesizable chemical compound libraries covering large portions of chemical space at hand (58). Furthermore, as GPCRs, which are *bona fide* targets for small-molecule drugs, FZDs have the conceptual potential to be accessible and druggable.

### Back to square one?

Targeting FZDs with small molecules has been and still is notoriously difficult and slow. The barriers to implementation might be attributed to several underlying factors. First of all, the signalosome concept, claiming that the sole mechanistic factor leading to WNT/β-catenin pathway initiation is FZD-LRP5/6 proximity, independent of GPCR-like dynamics, has been and is still standing in the way of drugging FZDs with small molecules pharmacologically and therapeutically (37, 38, 59, 60). Obvious evidence that FZDs indeed behave as dynamic pharmacological entities just like their cousins, the other approximately 800 GPCRs (3, 23, 39), must be embraced as a useful concept. While in the past, the lack of a class A GPCR-specific DRY motif in FZDs was used as an apparent weak argument that FZDs could not behave as GPCRs, we know now that FZDs contain a meaningful gear box of molecular switches important both for heterotrimeric G protein coupling as well as dynamic DVL association (17, 19, 23, 24). Furthermore, findings emerging from crystallographic studies on FZDs were not particularly encouraging, in that these studies revealed a unique transmembrane fold containing an extremely narrow and highly hydrophilic pocket, which led to the unproven and unvalidated conclusion that FZDs are not able to accommodate small molecules (61). On top of these conceptual challenges, hit identification, definition of the mode-of-action and compound validation poses an intrinsic challenge when tools and suitable assays are limited, or compounds show substantial assay interference in the absence of adequate counter assays and control experiments. Indeed, several small-molecule compounds that have been reported as FZD-targeting molecules were recently scrutinized. Most of them did not withstand thorough validation (62, 63). Here, an antagonistic FZD_7_-CRD-targeting peptide (Fz7-21) stood out, emphasizing that small-molecule antagonists acting at the orthosteric site of FZDs present a viable concept (64).

### Previously unrecognized mechanisms of transduction

To target a seven transmembrane (7 TM) domain receptor with a particular architecture such as FZDs with small molecules, it is indeed advantageous to understand the mechanistic underpinnings of allosteric communication between the binding of the ligand (agonist) to the orthosteric binding site and the receptor-transducer interface. WNTs as the endogenous agonists for FZDs bind the CRD, which thus serves as the orthosteric binding site (21, 65). As seen in Figure 1*A*, the large lipoglycoproteins of the WNT family embrace the CRD in a way that can be compared with a hand pinching the target with a thumb and an index finger (21, 65). However, it is no understatement that the physical process of transmission of information from WNT-CRD binding to the intracellular receptor-transducer interface remains poorly understood. Macroscopically, agonist (WNT or Norrin) binding results in agonist-induced intramolecular conformational rearrangements in FZDs such as FZD dimer dynamics (66, 67, 68), CRD dynamics (31), FZD core conformational rearrangements (16, 32), FZD-DVL interface dynamics (19, 25, 33) and FZD-G protein coupling (10, 11, 12, 13, 14, 16, 17, 19, 69). Regarding the WNT/β-catenin pathway, WNT binding to FZDs simultaneously recruits the coreceptor LRP5/6 to form a signalosome and to establish higher order complexes required for efficient signaling (35, 36). It should be underlined that most of the above-listed dynamic WNT-induced conformational changes in FZDs do not require LRP5/6 action, which is conceptually important to understand the required integration of signalosome and dynamic activation model (25, 31, 32).

Interestingly, most class A GPCRs have their orthosteric binding site for small molecules in an accessible pocket within the 7 TM bundle of the receptor. Profiling of FZD structures by cryo-EM clearly indicate that FZDs contain a cavity in the center of the receptor, which could potentially serve as a ligand binding site. Until recently, it remained unclear, where a putative ligand would bind and how and if the ligand would be able to access the cavity, which is gated by a lid forming a dynamic bottleneck (34, 70). Furthermore, we need to better understand how small-molecule compounds that target the water-filled cavity in the 7 TM core of FZDs, affect the constitutive or agonist-induced activation of the receptor and the complex signaling paradigms that FZDs are involved in. By definition, a small molecule targeting the receptor core would not be an orthosteric ligand; it would instead be classified as an allosteric ligand or allosteric modulator. Conceptually, this is enormously important for the understanding of FZD activation mechanisms. The hypothesis that an allosteric modulator targets the core and acts for example as a negative allosteric modulator (NAM) can only be tested and confirmed if we embrace the concept of FZDs as dynamic gear boxes harboring GPCR-like behavior (3, 23, 39).

**Figure 2:**
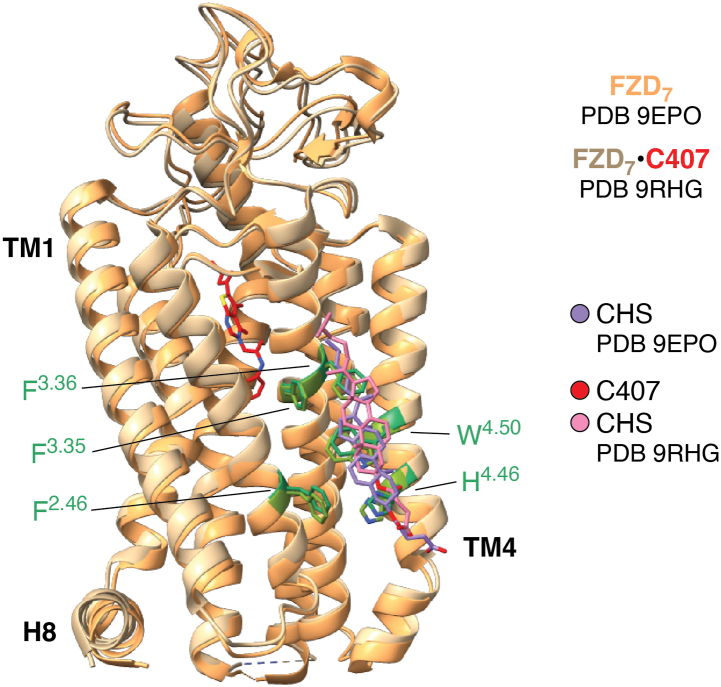
**Membrane cholesterol as an allosteric regulator of FZDs.** Overlay of two models of FZD_7_ in inactive conformations based on cryo-EM structure of FZD_7_ in the apo-form (PDB ID 9EPO; (34)) and in complex with C407 (PDB ID 9RHG; (70)), both resolved as an antiparallel dimer. The cholesterol (CHS) binding site is highlighted in green representing the family-wide conserved residues F^2.46^, F^3.35^, F^3.36^, W^4.50^ and H^4.46^. Mutation of the conserved CHS binding site abrogates the receptor’s ability to recruit the DEP domain of DVL emphasizing the allosteric coupling between the membrane cholesterol and the intracellular transducer interface (34).

Another exciting aspect underlying the relevance of protein dynamics and intramolecular allostery in FZDs is the recent discovery of a family-wide conserved cholesterol binding site between TM2, TM3 and TM4 on the outside of the 7TM barrel (34) (Fig. 2). Disruption of this conserved cholesterol binding site by mutagenesis interfered with constitutive (*i.e.* ligand-independent) DVL-DEP domain binding to FZD suggesting that the FZD-cholesterol interaction affects DEP binding allosterically. Indeed, this allosteric mechanism is highly reminiscent of the behavior of other GPCRs and therefore underlines the intrinsic dynamics required for FZD-mediated signal initiation. In connection with the discovery and pathway selectivity of state-stabilizing residues that act as molecular switches in FZDs, these opportunities for allosteric modulation open up possibilities for pathway selectivity of small-molecule modulators (17, 19, 23, 24).

### Conceptual basis for drugging FZDs with small molecules

Several obvious questions arise from the above-described findings. Can a FZD core-targeting NAM abrogate WNT-induced FZD/LRP5/6 complex-mediated signaling? Is the receptor sufficiently dynamic to procure access to the partially occluded, putative binding site deep in the FZD protein? In other words, despite the challenges, is the goal of developing more FZD-targeting small-molecule compounds achievable? (62, 63).

Compared to FZDs, SMO has a relatively rich pharmacology with small-molecule ligands of diverse efficacy ranging from agonists (SMO agonists or SAGs) to antagonists (SMO antagonists or SANTs) and inverse agonists, such as the natural alkaloid cyclopamine and derivatives (71, 72). In fact, small-molecule drugs targeting SMO are in clinical use for the treatment of basal cell carcinoma (73). Cholesterol/oxysterols present the natural, primary ligands for SMO targeting several distinct steroid binding sites in the receptor core as well as in the lipophilic groove of the CRD (4). Interestingly, despite the different nature of the agonists, evolution maintained the CRD in class F as a conserved structural and functional feature, a mechanistic conundrum that is not fully understood yet. Nevertheless, the similarity between SMO and FZDs is striking, and that was the basis for testing whether a SMO agonist such as SAG1.3 can act through FZDs, particularly by exploring FZD_6,_ the closest relative to SMO (74). Indeed, SAG1.3 acts at the seven transmembrane core of FZD_6_ as positive ago-allosteric modulator with weak positive efficacy compared to WNT-5A affecting the overall conformation of the FZD_6_ core, DVL interaction and heterotrimeric G_i_ protein coupling.

While repurposing SAG1.3 for FZDs was the very first step to target FZDs with small molecules, the questions remained whether the poor pharmacological characteristics of SAG1.3 could be improved by derivatization. In an attempt to explore the chemical space around SAG1.3, we synthesized a series of compounds, of which compound 11 (DJ503701) stood out (74, 75). Chemical modification of SAG1.3 (Fig. 1*B*) turned the ago-PAM into a NAM and compound 11 reduced both WNT-induced and WNT-surrogate-induced WNT/β-catenin signaling assessed by the luciferase-based TOPFlash reporter gene assay. Furthermore, compound 11 diminished WNT-induced conformational changes in the FZD-DVL DEP domain interface measured with the recently designed, BRET-based FZD-DEP-Clamp sensors (25, 33). These molecular assessments of NAM action go hand in hand with the compound’s ability to blunt WNT-3A-induced *LGR5* mRNA expression in spheroids derived from primary human hepatocytes and to reduce viability selectively in RNF43-negative pancreatic cancer cells (HPAF-II) but not in RNF43 wild-type cells (PANC-1). The latter is consistent with a FZD_5_-dependent signaling loop in pancreatic cancer cells carrying an RNF43 loss-of-function mutation (76). Thus, the overall characterization of compound 11 on WNT signaling presents a proof of concept since it appears to act as an NAM targeting the core of FZDs in a non-selective manner. Nevertheless, the exact binding site remains elusive, even though it seems likely, yet unproven, that compound 11 would occupy the same binding pocket as SAG1.3.

Mechanistically, it should also be emphasized that the core-targeting compound 11 not only blocked the WNT-3A-induced WNT/β-catenin signaling as assessed by the TOPFlash luciferase reporter assay, but that it also blunted the WNT surrogate-induced response (75). As mentioned above, WNT surrogates are designed to initiate WNT/β-catenin signaling by bringing FZDs and LRP5/6 in close proximity a characteristic of the signalosome model (77). If protein proximity, in the absence of FZD GPCR-like protein dynamics would be the sole driving force for initiating β-catenin signaling—as previously stated (38)—the NAM acting on the FZD core should not be able to block the WNT surrogate-elicited response in the TOPFlash assay. Nonetheless, the ability of a core-targeting FZD NAM to dramatically reduce the WNT surrogate-induced β-catenin signal, argues that intrinsic conformational rearrangements in FZDs are required to not only mediate WNT but also WNT surrogate effects. Thus, conformational dynamics must be considered as an integral part of the signalosome-mediated signal initiation (3).

**Figure 3:**
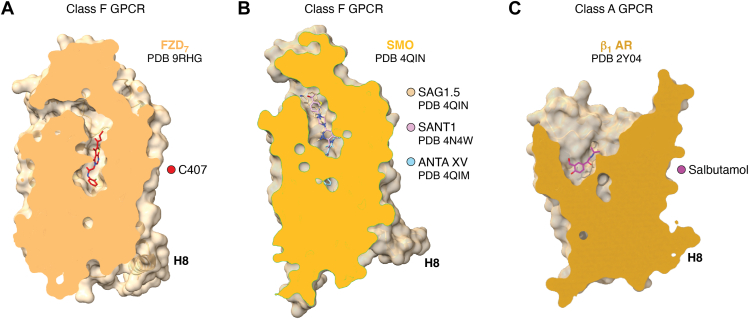
**Ligand binding sites in class F GPCRs compared to class A GPCRs.***A*, a model of FZD_7_ in complex with C407 is shown in side view slabbing the surface representation at the level of the central cavity (PDB ID 9RHG) (70). H8 – helix 8. *B*, model of SMO in complex with SAG1.5 (PDB ID 4QIN), SANT1 (PDB ID 4N4W) and ANTA XV (PDB ID 4QIM) is shown in side view slabbing the surface representation at the level of the central cavity (PDB ID 4QIN) (78). *C*, model of the turkey β_1_ adrenoceptor (β_1_AR) in complex with salbutamol is shown in side view slabbing the surface representation at the level of the orthosteric binding site (PDB ID 2Y04) (84).

In an orthogonal approach to find FZD-targeting small molecules, we employed large-scale virtual docking screens to several putative binding sites in the cavity within the 7TM domain of FZD_7_ (70). The emerging, most promising small-molecule compound C407 targets a deep pocket in the core of FZD_7_ corresponding to the SANT-1 binding site in SMO (78) (Figs. 2 and 3, *A* and *B*). While the compound C407 has a low potency in the micromolar range, its binding site could be experimentally confirmed employing orthogonal approaches such as site-directed mutagenesis, molecular dynamics (MD) simulations and cryo-EM (Fig. 2). The compound was characterized by its ability to reduce WNT-3A-induced FZD conformational changes as assessed by the FZD-DEP Clamp sensors and WNT/β-catenin signaling reported as TOPFlash signal. Indeed, the functional validation of C407 action on different FZD paralogs revealed poor selectivity for FZDs, which is further supported by the comparison of the overall conservation of residues in the proximity of the C407 binding site across the human paralogs (70). One of the key residues that shows polar interactions with C407 is the conserved Y^6.51^, which, among the 10 human FZDs, is conserved to 90% with FZD_8_ as the sole exception. C407 can be clearly identified as a NAM, and MD simulations of the receptor-ligand interactions revealed a putative mode of action for FZD-targeting NAMs. Throughout the simulation time, C407 interacted with and induced a conformational rearrangement of residues W^3.43^ and Y^6.40^ at the bottom of the binding cavity, which participate in the network of the extended molecular switch identified in class F receptors and involved in receptor activation (18, 24, 34). We hypothesize therefore, that deep binding of C407 and intercalation with the molecular switch network interferes with FZD activation to interrupt WNT-induced information transmission through the gear box of molecular switches in the receptor protein.

Compound 11 and C407 show micromolar potency. Future efforts will address whether it will be possible to optimize these compounds to increase affinity and potency. Ligands to SMO—in part in clinical use—present with higher affinity and potency in the nanomolar range. In a direct comparison of the overall architecture of the central ligand-accommodating cavity of class F GPCRs, it becomes obvious that the path through the receptor to reach the deep binding pocket, where, for example SANT1 and C407 bind, is complex, occluded and long (Fig. 3, *A* and *B*). Nevertheless, SMO pharmacology and experimental evidence for both SMO and FZD emphasize that the pocket is indeed accessible even in complex cellular contexts. Thus, despite a binding pocket architecture with apparent limited accessibility, class F GPCRs are accessible drug targets. The point of binding site accessibility becomes even more obvious when comparing class F GPCRs with a *bona fide* drug target from class A, such as the β_1_ adrenoceptor (Fig. 3*C*). The ligand binding pocket accommodating, for example, the partial agonist salbutamol is wide open and directly solvent accessible, enabling undisturbed binding and unbinding of compounds.

It should also be added that the CRD of class F GPCRs plays into the accessibility of the internal cavity and the binding pockets. While basal protein dynamics in the receptor core regulate accessibility of the core cavity, it remains unknown, whether WNT binding to the CRD affects access to and the overall architecture of the internal ligand binding cavity in FZDs (34). The role of the CRD for the binding of small-molecule ligands to the central cavity of SMO has actually been quantified by comparing full length SMO and ΔCRD-SMO employing a NanoBRET-based kinetic binding assay using BODIPY-cyclopamine as tracer (79). The quantitative binding data suggest a slightly higher on-rate of BODIPY-cyclopamine and a higher pK_i_ for unlabeled compounds in competition binding assays in the absence of the CRD implying that the presence of the CRD of SMO at least in part obstructs access to the ligand binding site in the core cavity. Furthermore, another aspect becomes visually obvious (Fig. 3*B*), when comparing the different SMO structures in complex with ligands, such as the agonist SAG1.5 and the antagonists/inverse agonists SANT1 and ANTA XV: almost the whole internal cavity of SMO is lined with individual binding sites for drug-like compounds. As a consequence, there is plenty of room for the exploration of FZD-targeting compounds along the central cavity in FZDs, with the potential to find ligands with positive, neutral or negative efficacy.

### Will it be possible to avoid unwanted side effects?

With the clinical failure of vantictumab/OMP-18R, a non-selective FZD antibody targeting the CRD and thereby interfering with WNT binding and WNT-induced signaling, it became apparent that the attraction of targeting FZDs therapeutically comes along with a substantial risk for unwanted side effects, such bone-related safety (55, 80, 81). Given the pivotal role of WNT/FZD-driven turnover of epithelium and the WNT/FZD-dependent regulation of bone formation, these processes are impeded by broad FZD-targeting compounds. This overhanging risk of severe side effects upon exposure to FZD NAMs can only be minimized by reducing systemic compound exposure and increasing FZD paralog-selectivity. The high degree of sequence conservation among the human FZD paralogs clearly poses a challenge to identifying FZD family member (paralog)-selective compounds. Similarity within the four FZD homology clusters composed of FZD_1, 2, 7_, FZD_3, 6_, FZD_5, 8_ and FZD_4, 9, 10_ opens also the realistic and attractive compromise to achieve cluster rather than paralog selectivity.

## Conclusions—light at the end of the tunnel

The recently reported compounds (compound 11 and C407) are suboptimal regarding potency and paralog selectivity, but they present a true breakthrough and a validated starting point for further investigations, compound identification and optimization. They suggest that there may be light at the end of the tunnel. During the identification of C407, another hit compound emerged from the first screen (C45). Although C45 similarity searches identified C407 as another FZD-targeting small molecule, C45, when used as a FZD-targeting compound, showed high off-target toxicity and was therefore discarded (70). Nevertheless, C45 might be useful as a reference standard for the purposes of control assays and counter assays in any screening and post-screening validation campaigns. The report of these assays that control for toxicity and assay interference of potential hit compounds is particularly relevant when employing luciferase-based assays (gene reporter assays (TOPFlash or similar) or BRET assays) as well as fluorescence-based approaches, since luciferase inhibitors or fluorescence quenchers notoriously interfere with these readouts (63, 82).

On the more positive side, the concept of targeting FZDs allosterically in their core and thereby achieving blockade of the WNT/β-catenin pathway is now established. Thus, GPCR-like receptor dynamics according to a WNT/FZD/DVL ternary complex model provide the basis for the compound mode of action as NAMs (15, 83). Nevertheless, affinity, potency and efficacy of the FZD-targeting NAMs must be improved, which in fact might be a challenge, given the accessibility of the central ligand-binding cavity. Furthermore, paralog selectivity or at least FZD homology cluster selectivity is desirable to be able to move forward towards clinical implications.

## Data availability

No data were generated or analysed.

## Conflict of interest

The author declares that he does not have any conflicts of interest with the content of this article.
